# Towards the synthetic design of camelina oil enriched in tailored acetyl-triacylglycerols with medium-chain fatty acids

**DOI:** 10.1093/jxb/ery225

**Published:** 2018-07-06

**Authors:** Sunil Bansal, Hae Jin Kim, GunNam Na, Megan E Hamilton, Edgar B Cahoon, Chaofu Lu, Timothy P Durrett

**Affiliations:** 1 Department of Biochemistry and Molecular Biophysics, Kansas State University, Manhattan, KS, USA; 2 Department of Biochemistry and Center for Plant Science Innovation, University of Nebraska-Lincoln, Lincoln, NE, USA; 3 Department of Plant Sciences and Plant Pathology, Montana State University, Bozeman, MT, USA; 4 Department of Chemistry and Biology, Bethany College, Lindsborg, KS, USA

**Keywords:** Acetyl-TAG, biofuels, bioproducts, *Camelina sativa*, medium chain fatty acid, synthetic biology, vegetable oil

## Abstract

The ability to manipulate expression of key biosynthetic enzymes has allowed the development of genetically modified plants that synthesise unusual lipids that are useful for biofuel and industrial applications. By taking advantage of the unique activities of enzymes from different species, tailored lipids with a targeted structure can be conceived. In this study we demonstrate the successful implementation of such an approach by metabolically engineering the oilseed crop *Camelina sativa* to produce 3-acetyl-1,2-diacyl-*sn*-glycerols (acetyl-TAGs) with medium-chain fatty acids (MCFAs). Different transgenic camelina lines that had been genetically modified to produce MCFAs through the expression of MCFA-specific thioesterases and acyltransferases were retransformed with the *Euonymus alatus* gene for diacylglycerol acetyltransferase (EaDAcT) that synthesises acetyl-TAGs. Concomitant RNAi suppression of acyl-CoA:diacylglycerol acyltransferase increased the levels of acetyl-TAG, with up to 77 mole percent in the best lines. However, the total oil content was reduced. Analysis of the composition of the acetyl-TAG molecular species using electrospray ionisation mass spectrometry demonstrated the successful synthesis of acetyl-TAG containing MCFAs. Field growth of high-yielding plants generated enough oil for quantification of viscosity. As part of an ongoing design–test–learn cycle, these results, which include not only the synthesis of ‘designer’ lipids but also their functional analysis, will lead to the future production of such molecules tailored for specific applications.

## Teaser

Plants of the oilseed crop camelina were metabolically engineered to produce acetyl-triacylglycerol molecules containing medium-chain fatty acids, resulting in tailored lipids with a designed structure that are potentially useful for biofuel and industrial applications.

## Introduction

**Fig. 1.**
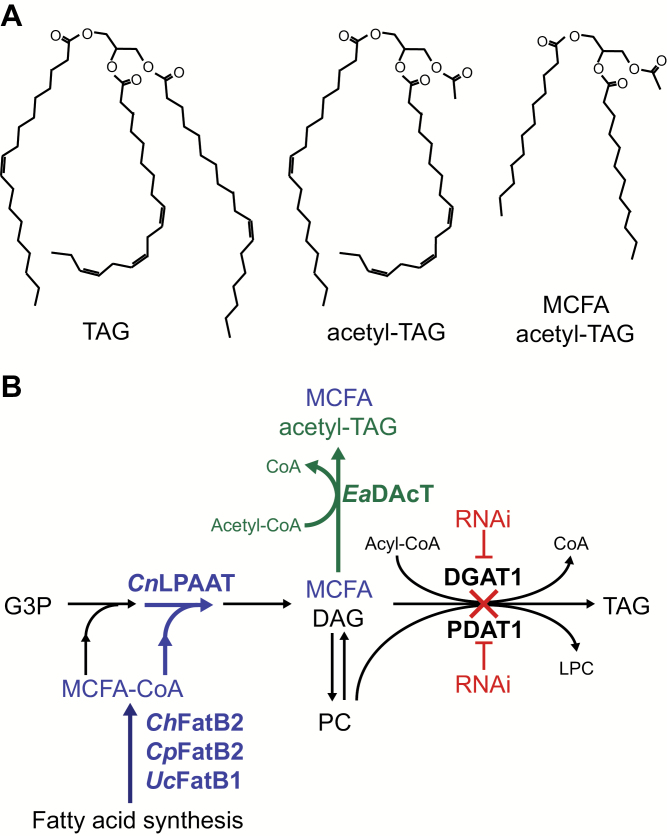
Strategy for the production of MCFA acetyl-TAGs in transgenic oilseeds. (A) Structures of representative TAG, acetyl-TAG, and acetyl-TAG containing MCFA. (B) Metabolic pathways leading to the synthesis of acetyl-TAGs containing MCFAs. Compound abbreviations: acetyl-TAG, 3-acetyl-1,2-diacyl-*sn*-glycerol; CoA, co-enzyme A; DAG, diacylglycerol; G3P, glycerol-3-phosphate; LPC, lysophosphatidylcholine; MCFA, medium chain fatty acid; PC, phosphatidylcholine; TAG, triacylglycerol. Enzyme abbreviations: DAcT, diacylglycerol acetyltransferase; DGAT1, diacylglycerol acyltransferase; Fat, fatty acid thioesterase; LPAAT, lysophosphatic acid acyltransferase; PDAT1, phosphatidylcholine: diacylglycerol acyltransferase. Species abbreviations: *Ch*, *Cuphea hookeriana*; *Cn*, *Cocos nucifera*; *Cp*, *Cuphea palustris*; *Ea*, *Euonymus alatus*; *Uc*, *Umbellularia californica*.

Seed oils are comprised mostly of triacylglycerols (TAGs), energy-dense molecules that consist of three fatty acids esterified to a glycerol backbone (Fig. 1A). The fatty acid composition of the TAG molecular species determines the physical properties of a particular vegetable oil. TAGs from commercially grown oilseed crops typically contain mainly five fatty acids: palmitic, stearic, oleic, linoleic, and linolenic. In contrast, throughout the plant kingdom, a wide variety of fatty acids with different chain lengths and functional groups exist (Badami and Patil, 1980). The presence of these unusual fatty acids often imparts the seed oil with useful functionalities. Among them, medium-chain fatty acids (MCFAs) with chain lengths of 8–14 carbons can be used for the production of detergents, soaps, lubricants, and biofuels (Dyer *et al.*, 2008). MCFAs are mostly found in tropical plants such as coconut and palm kernel, but temperate plants such as the *Cuphea* genus (Graham, 1989) and the California bay laurel (*Umbellularia californica*) produce high levels of MCFAs in their seeds. Even shorter acyl groups are possible. Various Celestraceae species, such as *Euonymus alatus*, synthesise 3-acetyl-1,2-diacylglycerols (acetyl-TAGs), unusual TAGs with a two-carbon acetate group esterified to the *sn*-3 position (Kleiman *et al.*, 1967; Sidorov *et al.*, 2014; Tran *et al.*, 2017). The *sn*-3 acetate group means that acetyl-TAGs possess useful physical properties compared to TAGs with a long-chain fatty acid at that position (hereafter referred to as lcTAGs). For example, acetyl-TAGs possess a lower kinematic viscosity compared to lcTAGs, as well as improved cold-temperature properties, suggesting potential applications as improved biofuels and biolubricants (Durrett *et al.*, 2010; Liu *et al.*, 2015*a*, 2015*b*).

The identification of enzymes important for the synthesis and incorporation of unusual fatty acids has allowed their production in transgenic plants through the expression of the encoding genes. For example, the *E. alatus* diacylglycerol acetyltransferase (EaDAcT) is necessary and sufficient for the synthesis of the acetyl-TAGs that accumulate in the seed endosperm and embryo of that species (Durrett *et al.*, 2010). The seed-specific expression of *EaDAcT* resulted in acetyl-TAG levels ranging from 47–64 mole percent (mol%) in transgenic *Camelina sativa* lines. Further, *EaDAcT* expression combined with the suppression of the enzymes for lcTAG synthesis, e.g. diacylglycerol acyltransferase (DGAT1) and phospholipid diacylglycerol acyltransferase (PDAT1), resulted in acetyl-TAG levels as high as 85 mol% in the best transgenic lines (Liu *et al.*, 2015*a*, 2015*b*).

Similarly, MCFAs have been synthesised in a variety of transgenic oilseed crops, through the expression of specialised FatB acyl-ACP thioesterases that cause the release of nascent fatty acids before additional cycles of fatty acid synthesis can extend their carbon chain lengths to C16 or longer (Pollard *et al.*, 1991). In a number of different studies, the seed-specific expression of such specialised thioesterases from plants such as the California bay laurel or different *Cuphea* species resulted in the accumulation of MCFAs in transgenic canola (*Brassica napus*) seed (Dehesh *et al.*, 1996; Voelker *et al.*, 1996). Additional MCFA-specific FatB thioesterases were identified in *C. viscosissima* and *C. palustris* (Tjellström *et al.*, 2013; Kim *et al.*, 2015*b*). Subsequent expression of individual or different combinations of these specialised FatB thioesterases in camelina resulted in a range of fatty acid combinations of 8:0 to 16:0 in the transgenic seed oil (Kim *et al.*, 2015*b*; where, for example, 8:0 refers to a fatty acid with 8 carbon atoms and 0 double bonds). However, accumulation of MCFAs in transgenic seeds expressing these thioesterases was much lower compared to the levels present in the species that naturally synthesise these unusual fatty acids. The co-expression of a MCFA-specific lysophosphatic acid acyltransferase (LPAAT) from coconut was successful in increasing laurate accumulation at the *sn*-2 position and thus the overall levels of 12:0 (Knutzon *et al.*, 1999). Similarly, expression of other MCFA-specific LPAATs from *C. viscosissima* and *C. pulcherrima* caused the accumulation of MCFAs at the *sn*-2 position in transgenic camelina expressing various *Cuphea* FatB thioesterases (Kim *et al.*, 2015*a*).

The oilseed crop *Camelina sativa* has emerged as a useful platform for the synthesis of unusual lipids through biotechnology approaches. Importantly, a modified floral dip transformation method (Lu and Kang, 2008) enables the relatively easy development of different transgenic lines, allowing optimisation of transgene combinations. Multiple selectable markers permit the stacking of different transgenic traits through crossing or retransformation (Shockey *et al.*, 2015). In addition, field growth of transgenic camelina plants has allowed the large-scale production and subsequent functional testing of different lipids resulting from metabolic engineering, including acetyl-TAGs (Liu *et al.*, 2015*a*, 2015*b*), omega-7 fatty acids (Nguyen *et al.*, 2015), and the nutritionally important omega-3 very long-chain polyunsaturated fatty acids eicosapentaenoic acid (EPA) and docosahexaenoic acid (DHA) (Ruiz-Lopez *et al.*, 2014; Betancor *et al.*, 2015, 2016).

Here, we describe a synthetic-biology approach to metabolically engineer camelina to produce tailored lipid molecules with a designed structure. By combining the expression of enzymes resulting in the production of MCFAs with those resulting in high levels of acetyl-TAGs, we demonstrate the synthesis of MCFA-containing acetyl-TAGs that have not been found in nature (Fig. 1). Further, some of the transgenic camelina lines were grown in the field to produce these oils for functional testing of physical properties and they provided information to guide subsequent modifications of the lipid structure. Such iterative approaches will lead to targeted production of lipid molecules designed for specific applications.

## Materials and methods

### Plant transformation

Plasmids expressing *EaDAcT* alone or in combination with *DGAT1* RNAi and/or *PDAT1* RNAi (Liu *et al.*, 2015*a*) were modified by replacement of the *DsRed* selectable marker with the *BAR* gene that provides resistance to glufosinate. The gene, along with its nopaline synthase promoter, was amplified from the plasmid pBinGlyBar1 (Nguyen *et al.*, 2013) using primers with the sequences 5′-GCAGAGCTCGATCTCGGTGAC GGGCAGGACCGGA-3′ and 5′-AGCGATCGGCACGCTGCCGCAAGCACTCAGGGC -3′. The amplified product was digested with *Sac*I and *Pvu*I and ligated into the corresponding sites of the binary vectors containing *EaDAcT*, a process that simultaneously removed the *DsRed* gene and promoter cassette. The resulting binary vectors (see Supplementary Fig. S1 at JXB online) were introduced into *Agrobacterium tumefaciens* GV3101 and transformed into the MCFA-producing camelina lines *ChFatB2*, *ChFatB2*+*CpFatB2*, *UcFatB1*+*CnLPAAT* and *CpFatB2*+*UcFatB1* (Kim *et al.*, 2015*b*) using a floral vacuum-infiltration method (Lu and Kang, 2008). T_1_ plants were selected by spraying young seedlings four times with 0.01% (w/v) glufosinate. Lines containing one transgenic locus were identified based on a 3:1 segregation ratio for glufosinate resistance in the T_2_ generation. Independent lines with high levels of acetyl-TAGs were further propagated in the greenhouse to generate T_4_ seed. Large amounts of T_5_ seed for oil property analysis were harvested from plants grown at the Montana State University A. H. Post agricultural research farm near Bozeman, MT, under guidelines of the Animal and Plant Health Inspection Service, United States Department of Agriculture (USDA APHIS) permit no. BRS 16-032-106r.

### Lipid analysis

Seed lipids were extracted using a modified hexane-isopropanol extraction method (Li *et al.*, 2006) with tripentadecanoin (Nu Check Prep, Waterville, MN) added as an internal standard. Acetyl-TAGs and lcTAGs were quantified by separating those fractions using TLC, followed by transmethylation and gas chromatography as described previously (Liu *et al.*, 2015*a*). For electrospray ionisation mass spectrometry (ESI-MS) analysis, neutral lipids were isolated by elution from total seed lipid extracts through a small silica column with 99:1 (v/v) chloroform:methanol. Samples were directly infused into an API4000 Triple Quadrupole mass spectrometer (Applied Biosystems) using methods described previously (Bansal and Durrett, 2016*b*). The total oil content for different lines was determined gravimetrically on oil extracts from 100 mg of T_4_ seed.

### Positional analysis of TAGs

The *sn*-2 fatty acid composition of acetyl-TAGs and lcTAGs in T_4_ seed oil were determined by using the lipase from *Thermomyces lanuginosus* (Sigma-Aldrich), which specifically cleaves the *sn*-1 and -3 ester bonds of TAGs to generate 2-monoacylglycerols (2-MAGs). Samples of TLC-purified acetyl-TAGs or lcTAGs (1.5 mg) were dissolved in 1.0 ml of diethyl ether, after which 5000 U of lipase in 1 ml of 50 mM Tris buffer (pH 7.2) was added. The reaction was incubated at 37 °C for 30 min with constant shaking. Lipids were removed by extracting twice with 3 ml of diethyl ether and separated on boric acid-impregnated silica gel TLC plates using a chloroform:acetone solvent system (80:10, v/v). Next, 10 µg of tripentadecanoin was added to the bands corresponding to 2-MAGs, which were then scraped and the lipids extracted using 2 ml of toluene. After transmethylation using a base-catalysed reaction, the resulting fatty acid methyl esters were quantified using gas chromatography.

### Viscosity analyses

Oil was extracted from batches of 30–50 g of T_5_ seed by finely granulating in a coffee grinder followed by Soxhlet extraction with hexane. Acetyl-TAGs and lcTAGs were purified using silica gel chromatography in a 64-mm diameter glass column. Approximately 20 g of oil was applied to 400 g of silica; lipids were eluted with a step gradient that changed the proportions of hexane:diethyl ether from 100:0 to 95:5, to 90:10, to 80:20, and finally to 70:30 (v/v). Fractions (50 ml) were tested for purity using TLC with a 70:30:1 (v/v/v) hexane:diethyl ether:acetic acid solvent system. The fractions containing either pure acetyl-TAGs or lcTAGs were combined and the solvent removed using a rotary evaporator. This extraction and purification process was repeated to obtain enough acetyl-TAGs and lcTAGs from each background to quantify viscosity, typically 25–30 ml. The purity of the fractions was confirmed using ESI-MS. The kinematic viscosity of the purified acetyl-TAGs and lcTAGs was measured using a calibrated Ubbelohde viscometer (Cannon Instruments, State College, PA) at 40 °C according to the ASTM D445 method (ASTM International, 2017).

## Results and discussion

### Synthesis of acetyl-TAGs in MCFA-producing camelina lines

**Fig. 2.**
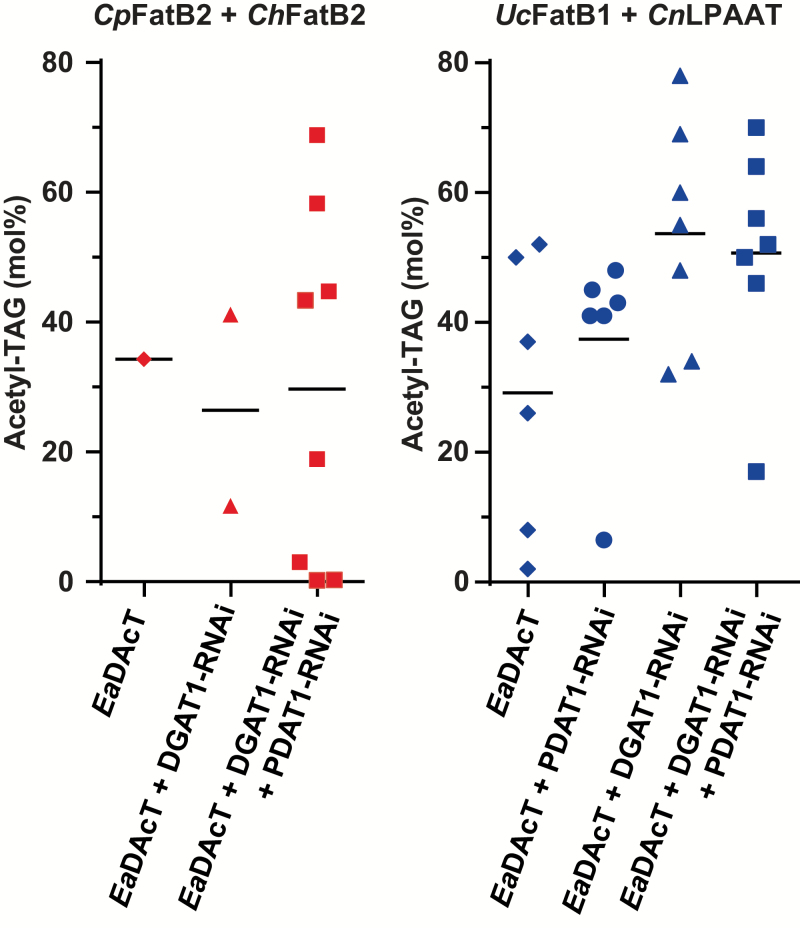
Expression of *EaDAcT* combined with down-regulation of *DGAT1* enhances acetyl-TAG accumulation. Scatter plots of the distribution of acetyl-TAG composition of homozygous T_3_ seeds from independent camelina lines expressing *CpFatB2* and *ChFatB2* or *UcFatB1* and *CnLPAAT*, and transformed with *EaDAcT* alone or in combination with RNAi constructs targeting camelina *DGAT1* and *PDAT1* homeologues. Horizontal lines represent the mean values for each sample group.

To produce acetyl-TAGs containing MCFAs *in planta*, we introduced *EaDAcT* into four different camelina backgrounds, *ChFatB2*, *CpFatB2*+*UcFatB1*, *CpFatB2*+*ChFatB2*, and *UcFatB1*+*CnLPAAT*, which had been previously engineered to produce MCFAs (Kim *et al.*, 2015*b*). We also combined the expression of *EaDAcT* with *DGAT1*-RNAi and *PDAT1*-RNAi constructs targeting the assembly of lcTAGs, which have previously been shown to increase the accumulation of acetyl-TAGs (Liu *et al.*, 2015*a*, 2015*b*). We found that the transformation efficiency of the MCFA-producing lines was often low; consequently, we only obtained one or two independent lines per combination of plant genotype and transformation vector. For example, only a total of five *EaDAcT*-stacked lines were obtained in the *ChFatB2* background and just three stacked lines in the *CpFatB2*+*UcFatB1* background (Supplementary Fig. S2). Slightly more lines were obtained when transforming the *CpFatB2*+*ChFatB2* genotype, particularly with the *EaDAcT* construct that also targeted both *DGAT1* and *PDAT1* (Fig. 2). Quantification of acetyl-TAGs in homozygous T_3_ seeds revealed a wide range of accumulation levels, with one line producing around 70 mol% acetyl-TAGs whereas three other lines accumulated no or low amounts. Similar bimodal results for acetyl-TAG accumulation have been observed before when expressing *EaDAcT* in Arabidopsis (Liu *et al.*, 2015*a*); the reason for this phenomenon remains unknown. In contrast to the other three MCFA backgrounds, multiple transgenic lines were obtained in the *UcFatB1*+*CnLPAAT* background with all *EaDAcT*-expressing vectors (Fig. 2). Transformation with *EaDAcT* alone resulted in acetyl-TAG levels of around 50 mol% in the T_3_ seed of the best transgenic lines, within range of previous observations in a wild-type (WT) background (Liu *et al.*, 2015*a*). Combining *EaDAcT* expression with the suppression of *PDAT1* did not result in higher levels of acetyl-TAGs. In contrast, co-expression of *EaDAcT* with *DGAT1*-RNAi led to higher levels of acetyl-TAGs, with approximately 77 mol% in the best line. The combination of *EaDAcT* expression with RNAi of both *DGAT1* and *PDAT1* failed to increase these levels further. These results were consistent with our previous work in a WT background where we observed that the addition of an RNAi construct targeting *PDAT1* failed to enhance acetyl-TAG levels beyond those observed with just *DGAT1*-RNAi (Liu *et al.*, 2015*a*). One potential explanation is that because both genes have been shown to be important for seed development in Arabidopsis (Zhang *et al.*, 2009), the concomitant strong down-regulation of both *DGAT1* and *PDAT1* is likely harmful to camelina embryo development.

### EaDAcT can synthesise MCFA acetyl-TAGs in camelina

**Fig. 3.**
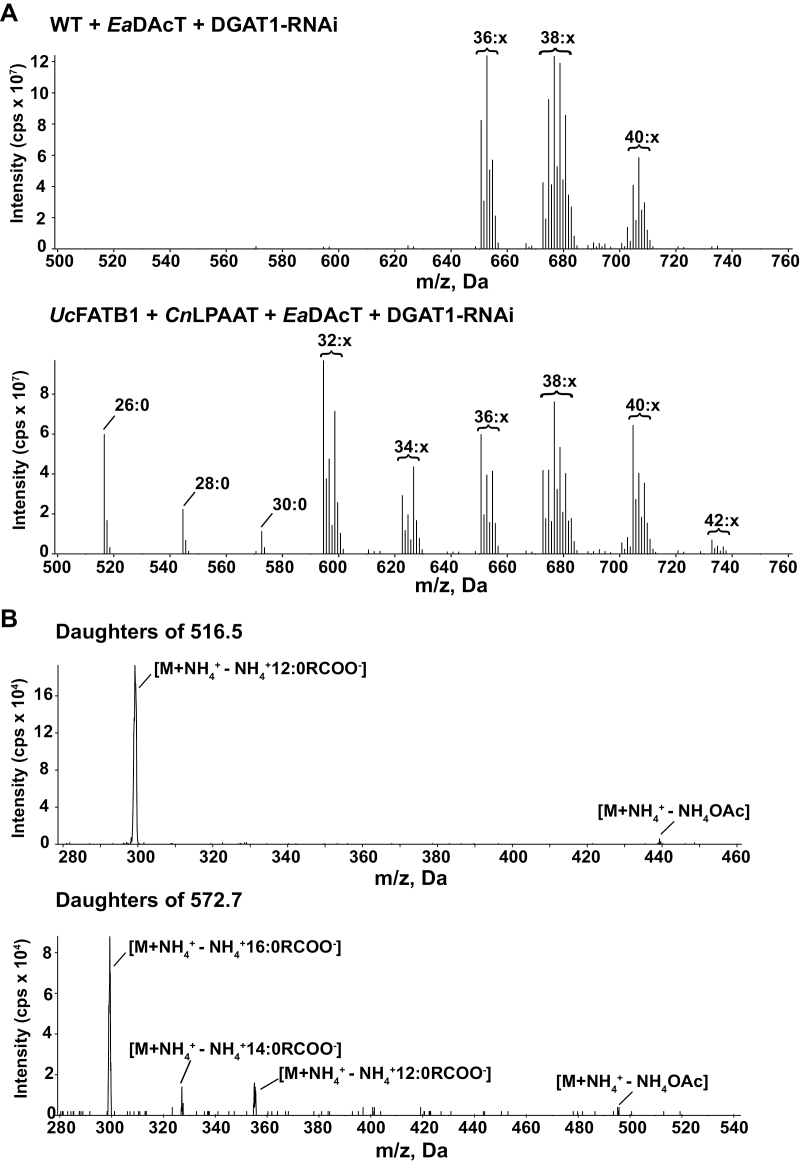
Synthesis of medium chain acetyl-TAGs in camelina. (A) Positive electrospray ionisation tandem mass spectrometry (ESI-MS^2^) spectra scanning for the neutral loss of ammonium acetate to detect acetyl-TAG molecular species in neutral lipid extracts from T_3_ homozygous seeds of wild-type (WT) and MCFA-producing camelina plants expressing *EaDAcT*. Signal peaks possess the *m*/*z* value of the [M+NH_4_]^+^ adduct. For clarity, only the number of acyl carbons and not the number of double bonds (x) in each series of acetyl-TAG molecular species is indicated. (B) ESI-MS^2^ daughter scans of medium-chain acetyl-TAGs from camelina seed expressing *EaDAcT*. The data shown are the fragment peaks for acetyl-TAGs with [M+NH_4_]^+^ adducts with masses of 516.5 and 572.7.

We analysed the composition of neutral lipids extracted from the seeds of homozygous T_3_ plants using ESI-MS neutral loss scans to identify acetyl-TAG molecular species. The spectra of MCFA-producing lines expressing *EaDAcT* contained novel lower molecular-mass acetyl-TAG peaks compared to a WT background line expressing *EaDAcT* (Fig. 3A). The mass of these peaks corresponded to acetyl-TAG molecules containing MCFA. For example, the ammonium adduct of acetyl-dilaurin (indicated by 26:0 in Fig. 3A) causes the novel peak with *m*/*z* of 516.5. The presence of MCFAs in these lower molecular-mass acetyl-TAGs was further confirmed using ESI-tandem MS (ESI-MS^2^), which produced daughter fragments indicative of MCFAs (Fig. 3B). Together, these results demonstrated that EaDAcT was able to acetylate MCFA diacylglycerols (DAGs) such as 1,2-dilaurin-*sn*-3-glycerol and 1,2-dimyristoyl-*sn*-3-glycerol to generate acetyl-dilaurin and acetyl-dimyristin in camelina seed. Such activities have been shown *in vitro* in our previous work (Bansal and Durrett, 2016*a*).

**Fig. 4.**
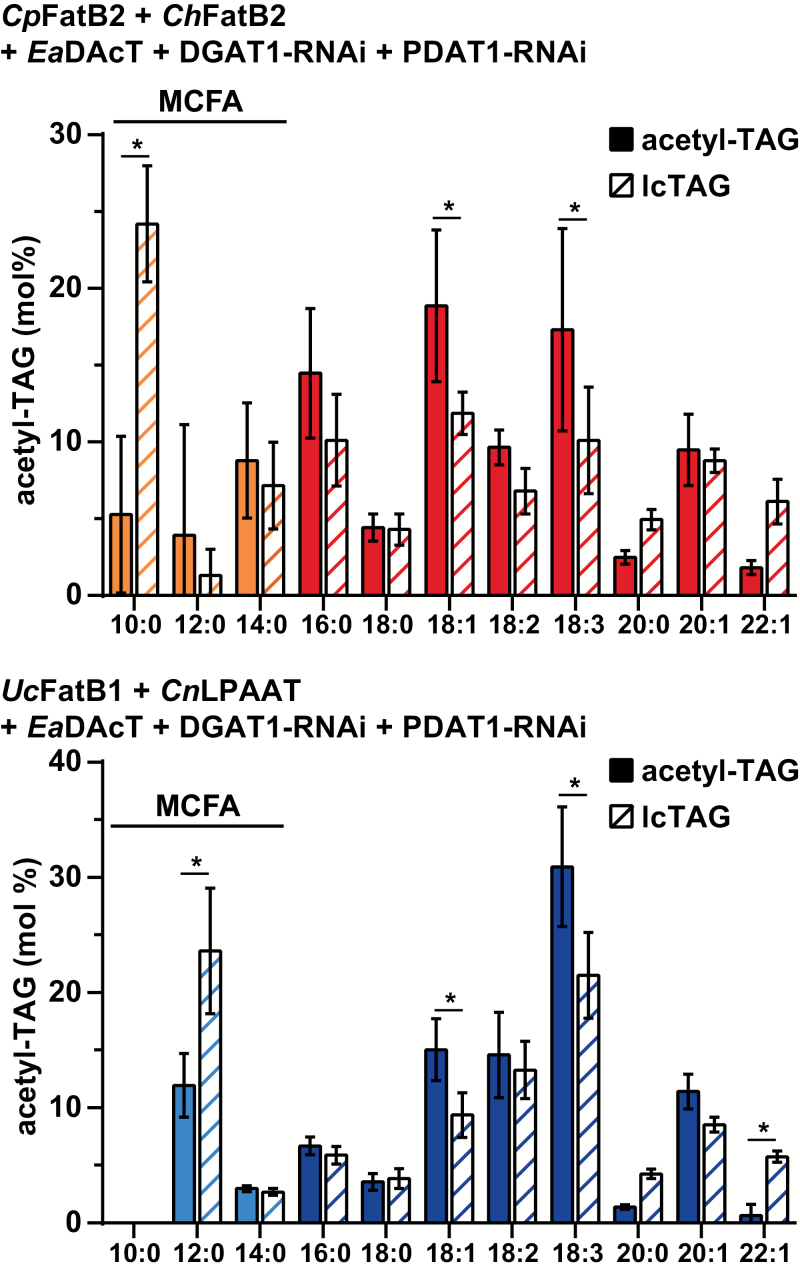
Acetyl-TAGs contain lower levels of MCFAs than lcTAGs. Mean fatty acid composition of acetyl-TAGs and lcTAG fractions from T_3_ seed of independent homozygous lines in the *CpFatB2*+*ChFatB2* (*n*=4) or *UcFatB1*+*CnLPAAT* (*n*=6) backgrounds expressing *EaDAcT* and RNAi cassettes targeting *DGAT1* and *PDAT1*. Data are means (±SD). Significant differences as determined by Student’s *t*-test with Holm–Šidák correction method for multiple comparisons are indicated: *P<0.05.

We also quantified the fatty acid composition of the acetyl-TAG and lcTAG fractions of high acetyl-TAG-producing lines in the *CpFatB2*+*ChFatB2* and the *UcFatB1*+*CnLPAAT* backgrounds. The distribution of the longer fatty acids typically found in wild-type camelina was generally consistent with previous observations (Liu *et al.*, 2015*a*): acetyl-TAGs were enriched in more unsaturated fatty acids such as 18:3 and possessed lower amounts of very long-chain fatty acids compared to lcTAGs (Fig. 4). However, in both genotypes, acetyl-TAGs possessed lower amounts of MCFAs compared to the lcTAGs produced in the same lines. For example, in the *CpFatB2*+*ChFatB2* background, 10:0 levels were about four-fold lower in acetyl-TAGs compared to lcTAGs. Likewise, in the *UcFatB1*+*CnLPAAT* lines, 12:0 levels in acetyl-TAGs were on average half those in lcTAGs (Fig. 4).

### MCFAs are inefficiently incorporated at the *sn*-2 position of acetyl-TAGs

**Fig. 5.**
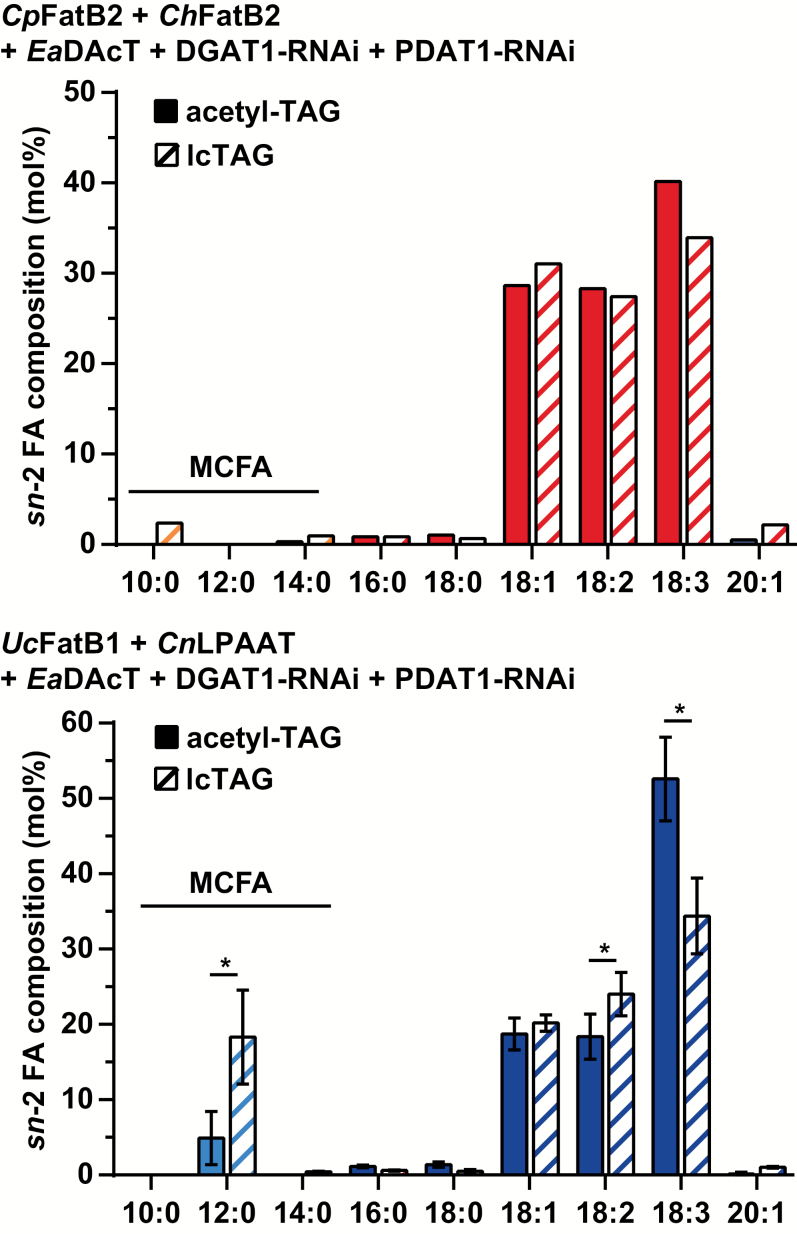
Acetyl-TAGs possess low incorporation of MCFAs at *sn*-2. Mean fatty acid composition at *sn*-2 positions of lcTAGs and acetyl-TAGs from T_4_ seed of four independent homozygous camelina *UcFatB1*+*CnLPAAT* lines expressing *EaDAcT* in combination with *DGAT1*-RNAi, and of one independent homozygous camelina *CpFatB2*+*ChFatB* line expressing *EaDAcT* in combination with suppression of *DGAT1* and *PDAT1*. Data are means (±SD). Significant differences as determined by Student’s *t*-test with Holm–Šidák correction method for multiple comparisons are indicated: *P<0.05.

As the *sn*-3 position of acetyl-TAG is occupied by an acetate group, the incorporation of MCFAs at the other two positions is particularly important to achieve high levels of these fatty acids in acetyl-TAGs. The regio-specific incorporation of MCFAs into lcTAGs and acetyl-TAGs in high acetyl-TAG-producing transgenic lines was therefore quantified through the use of a stereospecific lipase. Consistent with previous results (Kim *et al.*, 2015*b*), lcTAGs in lines expressing *CnLPAAT* contained ~20 mol% laurate at the *sn*-2 position whereas plants lacking this MCFA-specific LPAAT possessed minimal levels of MCFAs at this position (Fig. 5). Likewise, only acetyl-TAGs from lines expressing *CnLPAAT* possessed MCFAs at the *sn*-2 position. However, the levels of laurate at the *sn*-2 position of acetyl-TAGs were only 5 mol%, considerably lower than those in lcTAGs produced in the same lines (Fig. 5). Taken together, these results emphasise the importance of a MCFA-specific LPAAT to increase accumulation of these unusual fatty acids at the *sn*-2 position of storage lipids in engineered oilseeds (Knutzon *et al.*, 1999; Kim *et al.*, 2015*a*, 2015*b*). Further, the much lower incorporation of MCFAs in the *sn*-2 position of acetyl-TAGs relative to lcTAGs also suggests discrimination of EaDAcT relative to endogenous DGAT1 activity for MCFA-containing DAG substrates. This observation was consistent with earlier work where we demonstrated *in vitro* that EaDAcT preferentially acetylates DAG molecules containing unsaturated DAGs (Bansal and Durrett, 2016*a*).

### Transgenic lines possess a lower seed oil content

**Fig. 6.**
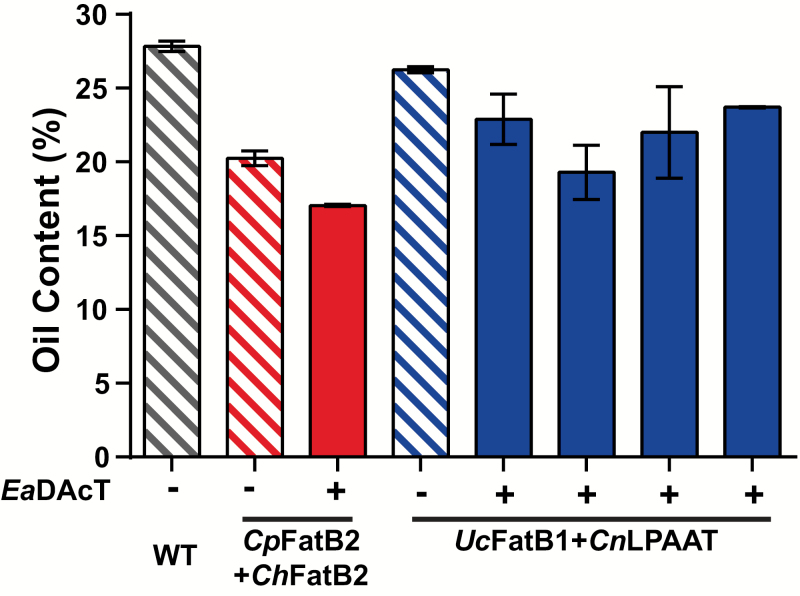
MCFA acetyl-TAG lines possess a lower oil content. Mean oil content of T_4_ seed of independent homozygous lines in the *CpFatB2*+*ChFatB2* and *UcFatB1*+*CnLPAAT* backgrounds expressing *EaDAcT* and *DGAT1*-RNAi. Data are means (±SD) of two independent gravimetric experiments.

When determined gravimetrically, the values for seed oil content of the MCFA-producing lines were lower than those of wild-type camelina. For *CpFatB2*+*ChFatB2* this reduction was quite large (73% of WT) whereas only a minimal reduction was observed with *UcFatB1*+*CnLPAAT* (Fig. 6). Similar reductions have been noted for other MCFA-producing camelina lines (Iskandarov *et al.*, 2017). The oil content of acetyl-TAG-producing lines derived from these two backgrounds was further reduced by 10–26%, depending on the specific line (Fig. 6). Previous work has shown that acetyl-TAG-producing lines in a WT background also possess slightly lower oil content (Liu *et al.*, 2015*a*). Interestingly, expression of a MCFA-specific DGAT1 has been shown to rescue the reduced oil content of camelina lines producing MCFAs (Iskandarov *et al.*, 2017). Here, the development of *EaDAcT* variants with improved specificity for MCFAs containing DAG might be helpful in overcoming the reduced oil content of these lines. Indeed, there are multiple reports of specialised transferases being able to reverse the reduced oil content caused by the production of unusual fatty acids in transgenic seeds (van Erp *et al.*, 2011; Hu *et al.*, 2012; Li *et al.*, 2012). An alternative strategy could be to overexpress the regulatory factor WRINKLED1 (WRI1), which rescues the reduced oil accumulation in Arabidopsis plants producing hydroxy fatty acids (Adhikari *et al.*, 2016).

### Viscosity of MCFA acetyl-TAGs

The viscosity of vegetable oils is an important parameter for their use as fuel, biodegradable lubricant, and other industrial applications. Previously, we demonstrated that acetyl-TAGs possess a lower kinematic viscosity compared to the lcTAGs found in most vegetable oils (Durrett *et al.*, 2010; Liu *et al.*, 2015*a*, 2015*b*). Similarly, other studies have shown that TAGs with MCFAs also possess lower viscosities compared to TAGs with longer chains (Valeri and Meirelles, 1997; Geller and Goodrum, 2000). To determine whether acetyl-TAGs enriched with MCFAs would possess lower viscosity than acetyl-TAGs with regular chain lengths, T_4_ seeds from two high acetyl-TAG-yielding lines from different MCFA background lines were bulked in the greenhouse and the field to yield enough oil for viscosity testing. The oil was subsequently fractionated using large-scale column chromatography to obtain pure acetyl-TAGs and lcTAGs.

The kinematic viscosities (measured at 40 °C according to ASTM D455) of acetyl-TAGs and lcTAGs from wild-type camelina plants were consistent with previous reports (Liu *et al.*, 2015*a*, 2015*b*), with acetyl-TAGs showing a ~30% reduction compared to lcTAGs (Table 1). LcTAGs from *UcFatB1*+*CnLPAAT* possessed a slightly lower viscosity of 29.4 mm^2^ s^–1^ than lcTAGs from wild-type plants (30.7 mm^2^ s^–1^). However, the viscosity of acetyl-TAGs synthesised in the *UcFatB1*+*CnLPAAT* background was essentially the same as that from a wild-type background, with both being 21.9 mm^2^ s^–1^. Interestingly, lcTAGs from *CpFatB2*+*ChFatB2* possessed a higher viscosity of 34.5 mm^2^ s^–1^ compared to wild-type lcTAGs. Likewise, the kinematic viscosity of acetyl-TAGs from this background was 23.8 mm^2^ s^–1^, higher than that of acetyl-TAGs synthesised in a wild-type background (Table 1).

**Table 1.** Kinematic viscosity (at 40 °C) of purified acetyl-TAGs and lcTAGs

Background	TAGs	Kinematic viscosity (mm^2^ s^–1^)^a^
Wild-type	lcTAGs	30.7 ± 0.02
	acetyl-TAGs^b^	21.9 ± 0.4
*CpFatB2*+*ChFatB2*	lcTAGs	34.5 ± 0.5
	acetyl-TAGs^c^	23.8 ± 0.03
*UcFatB1*+*CnLPAAT*	lcTAGs	29.4 ± 0.1
	acetyl-TAGs^b^	21.9 ± 0.06

^a^ Data represent means (±SD) for five replicate assays

^b^ From seed also expressing *EaDAcT* + *DGAT1*-RNAi

^c^ From seed also expressing *EaDAcT* + *DGAT1*-RNAi + *PDAT1*-RNAi

The unchanged or elevated viscosities of acetyl-TAGs containing MCFAs from *UcFatB1*+*CnLPAAT* or *CpFatB2*+*ChFatB2* backgrounds, respectively, probably reflects the poor incorporation of MCFAs into these molecules. Initial attempts to enrich the acetyl-TAG fraction for molecules containing MCFAs were unsuccessful. Another, non-exclusive possibility, particularly for the TAGs from the *CpFatB2*+*ChFatB2* background, is that the reduced polyunsaturated fatty acid (PUFA) levels in the acetyl-TAG and lcTAG fractions also contribute to the increase in viscosity. Previous work has shown that double bonds greatly reduce the kinematic viscosity of lcTAGs (Knothe and Steidley, 2005). Likewise, acetyl-TAGs produced in a camelina high-oleic background possess a higher viscosity than acetyl-TAGs containing more PUFAs from a wild-type background (Liu *et al.*, 2015*b*).

## Conclusions

By taking advantage of the combinatorial nature of synthetic biology we have successfully generated tailored lipid molecules not found in nature. However, additional metabolic engineering is needed to improve the incorporation of MCFAs into acetyl-TAGs to further alter the physical properties of these molecules. The recent availability of alternative MCFA-specific LPAAT enzymes (Kim *et al.*, 2015*a*) offers the possibility to improve MCFA incorporation into the *sn*-2 position of DAGs and subsequently acetyl-TAGs. However, strategies beyond increasing MCFA content in DAGs will be required given the apparent discrimination of EaDAcT against saturated DAG molecular species (Fig. 4; Bansal and Durrett, 2016*a*). It might be possible to use directed molecular evolution approaches, similar to those used to identify high-activity DGAT1 enzymes (Siloto *et al.*, 2009), to select for EaDAcT variants with improved specificity for MCFA-containing DAG molecular species. Alternatively, producing unsaturated MCFAs to enable better utilisation by EaDAcT would also result in an increased production of lower molecular-mass TAG species. This latter strategy also has the advantage in that the desired product would be more unsaturated, likely improving the viscosity and cold-temperature properties. Thus, iterative design–test–learn cycles that involve not only increasing the synthesis of tailored lipid molecules in seed oils, but also testing their physical and chemical properties, will lead to the production of lipid molecules designed for specific applications.

## Supplementary data

Supplementary data are available at *JXB* online.

Fig. S1. Constructs used to express *EaDAcT* and suppress camelina acyltransferases.

Fig. S2. Expression of *EaDAcT* combined with down-regulation of *DGAT1* enhances acetyl-TAG accumulation.

Supplementary figures S1-S2

## References

[CIT0001] Adhikari ND, Bates PD, Browse J 2016 *WRINKLED1* rescues feedback inhibition of fatty acid synthesis in hydroxylase-expressing seeds. Plant Physiology 171, 179–191. 27208047 10.1104/pp.15.01906 PMC4854691

[CIT0002] **ASTM International** 2017 Standard test method for kinematic viscosity of transparent and opaque liquids (and calculation of dynamic viscosity). ASTM D445-17a. West Conshohocken, PA: ASTM International.

[CIT0003] Badami RC, Patil KB 1980 Structure and occurrence of unusual fatty acids in minor seed oils. Progress in Lipid Research 19, 119–153. 7033990 10.1016/0163-7827(80)90002-8

[CIT0004] Bansal S, Durrett TP 2016a Defining the extreme substrate specificity of *Euonymus alatus* diacylglycerol acetyltransferase, an unusual membrane-bound *O*-acyltransferase. Bioscience Reports 36, e00406. 10.1042/BSR20160277 PMC5100001 27688773

[CIT0005] Bansal S, Durrett TP 2016b Rapid quantification of low-viscosity acetyl-triacylglycerols using electrospray ionization mass spectrometry. Lipids 51, 1093–1102. 27497979 10.1007/s11745-016-4179-0 PMC5890426

[CIT0006] Betancor MB, Sprague M, Montero D, Usher S, Sayanova O, Campbell PJ, Napier JA, Caballero MJ, Izquierdo M, Tocher DR 2016 Replacement of marine fish oil with *de novo* omega-3 oils from transgenic *Camelina sativa* in feeds for gilthead sea bream (*Sparus aurata* L.). Lipids 51, 1171–1191. 27590240 10.1007/s11745-016-4191-4 PMC5418318

[CIT0007] Betancor MB, Sprague M, Usher S, Sayanova O, Campbell PJ, Napier JA, Tocher DR 2015 A nutritionally-enhanced oil from transgenic *Camelina sativa* effectively replaces fish oil as a source of eicosapentaenoic acid for fish. Scientific Reports 5, 8104. 25632018 10.1038/srep08104 PMC4309969

[CIT0008] Dehesh K, Jones A, Knutzon DS, Voelker TA 1996 Production of high levels of 8:0 and 10:0 fatty acids in transgenic canola by overexpression of *Ch FatB2*, a thioesterase cDNA from *Cuphea hookeriana*. The Plant Journal 9, 167–172. 8820604 10.1046/j.1365-313x.1996.09020167.x

[CIT0009] Durrett TP, McClosky DD, Tumaney AW, Elzinga DA, Ohlrogge J, Pollard M 2010 A distinct DGAT with *sn*-3 acetyltransferase activity that synthesizes unusual, reduced-viscosity oils in *Euonymus* and transgenic seeds. Proceedings of the National Academy of Sciences, USA 107, 9464–9469. 10.1073/pnas.1001707107 PMC2889089 20439724

[CIT0010] Dyer JM, Stymne S, Green AG, Carlsson AS 2008 High-value oils from plants. The Plant Journal 54, 640–655. 18476869 10.1111/j.1365-313X.2008.03430.x

[CIT0011] Geller D, Goodrum J 2000 Rheology of vegetable oil analogs and triglycerides. Journal of the American Oil Chemists’ Society 77, 111–114.

[CIT0012] Graham SA 1989 *Cuphea*: a new plant source of medium-chain fatty acids. Critical Reviews in Food Science and Nutrition 28, 139–173. 2653730 10.1080/10408398909527495

[CIT0013] Hu Z, Ren Z, Lu C 2012 The phosphatidylcholine diacylglycerol cholinephosphotransferase is required for efficient hydroxy fatty acid accumulation in transgenic Arabidopsis. Plant Physiology 158, 1944–1954. 22371508 10.1104/pp.111.192153 PMC3320197

[CIT0014] Iskandarov U, Silva JE, Kim HJ, Andersson M, Cahoon RE, Mockaitis K, Cahoon EB 2017 A specialized diacylglycerol acyltransferase contributes to the extreme medium-chain fatty acid content of *Cuphea* seed oil. Plant Physiology 174, 97–109. 28325847 10.1104/pp.16.01894 PMC5411140

[CIT0015] Kim HJ, Silva JE, Iskandarov U, Andersson M, Cahoon RE, Mockaitis K, Cahoon EB 2015a Structurally divergent lysophosphatidic acid acyltransferases with high selectivity for saturated medium chain fatty acids from *Cuphea* seeds. The Plant Journal 84, 1021–1033. 26505880 10.1111/tpj.13063

[CIT0016] Kim HJ, Silva JE, Vu HS, Mockaitis K, Nam JW, Cahoon EB 2015b Toward production of jet fuel functionality in oilseeds: identification of FatB acyl-acyl carrier protein thioesterases and evaluation of combinatorial expression strategies in *Camelina* seeds. Journal of Experimental Botany 66, 4251–4265. 25969557 10.1093/jxb/erv225 PMC4493788

[CIT0017] Kleiman R, Miller RW, Earle FR, Wolff IA 1967 (S)-1,2-diacyl-3-acetins: optically active triglycerides from *Euonymus verrucosus* seed oil. Lipids 2, 473–478. 17805790 10.1007/BF02533174

[CIT0018] Knothe G, Steidley KR 2005 Kinematic viscosity of biodiesel fuel components and related compounds. Influence of compound structure and comparison to petrodiesel fuel components. Fuel 84, 1059–1065.

[CIT0019] Knutzon DS, Hayes TR, Wyrick A, Xiong H, Maelor Davies H, Voelker TA 1999 Lysophosphatidic acid acyltransferase from coconut endosperm mediates the insertion of laurate at the *sn*-2 position of triacylglycerols in lauric rapeseed oil and can increase total laurate levels. Plant Physiology 120, 739–746. 10398708 10.1104/pp.120.3.739 PMC59311

[CIT0020] Li R, Yu K, Wu Y, Tateno M, Hatanaka T, Hildebrand DF 2012 *Vernonia* DGATs can complement the disrupted oil and protein metabolism in epoxygenase-expressing soybean seeds. Metabolic Engineering 14, 29–38. 22107928 10.1016/j.ymben.2011.11.004

[CIT0021] Li Y, Beisson F, Pollard M, Ohlrogge J 2006 Oil content of Arabidopsis seeds: the influence of seed anatomy, light and plant-to-plant variation. Phytochemistry 67, 904–915. 16600316 10.1016/j.phytochem.2006.02.015

[CIT0022] Liu J, Rice A, McGlew K, Shaw V, Park H, Clemente T, Pollard M, Ohlrogge J, Durrett TP 2015a Metabolic engineering of oilseed crops to produce high levels of novel acetyl glyceride oils with reduced viscosity, freezing point and calorific value. Plant Biotechnology Journal 13, 858–865. 25756355 10.1111/pbi.12325

[CIT0023] Liu J, Tjellström H, McGlew K, et al 2015b Field production, purification and analysis of high-oleic acetyl-triacylglycerols from transgenic *Camelina sativa*. Industrial Crops and Products 65, 259–268.

[CIT0024] Lu C, Kang J 2008 Generation of transgenic plants of a potential oilseed crop *Camelina sativa* by *Agrobacterium*-mediated transformation. Plant Cell Reports 27, 273–278. 17899095 10.1007/s00299-007-0454-0

[CIT0025] Nguyen HT, Park H, Koster KL, Cahoon RE, Nguyen HT, Shanklin J, Clemente TE, Cahoon EB 2015 Redirection of metabolic flux for high levels of omega-7 monounsaturated fatty acid accumulation in camelina seeds. Plant Biotechnology Journal 13, 38–50. 25065607 10.1111/pbi.12233

[CIT0026] Nguyen HT, Silva JE, Podicheti R, et al 2013 Camelina seed transcriptome: a tool for meal and oil improvement and translational research. Plant Biotechnology Journal 11, 759–769. 23551501 10.1111/pbi.12068

[CIT0027] Pollard MR, Anderson L, Fan C, Hawkins DJ, Davies HM 1991 A specific acyl-ACP thioesterase implicated in medium-chain fatty acid production in immature cotyledons of *Umbellularia californica*. Archives of Biochemistry and Biophysics 284, 306–312. 1989513 10.1016/0003-9861(91)90300-8

[CIT0028] Ruiz-Lopez N, Haslam RP, Napier JA, Sayanova O 2014 Successful high-level accumulation of fish oil omega-3 long-chain polyunsaturated fatty acids in a transgenic oilseed crop. The Plant Journal 77, 198–208. 24308505 10.1111/tpj.12378 PMC4253037

[CIT0029] Shockey J, Mason C, Gilbert M, Cao H, Li X, Cahoon E, Dyer J 2015 Development and analysis of a highly flexible multi-gene expression system for metabolic engineering in Arabidopsis seeds and other plant tissues. Plant Molecular Biology 89, 113–126. 26254605 10.1007/s11103-015-0355-5

[CIT0030] Sidorov RA, Zhukov AV, Pchelkin VP, Vereshchagin AG, Tsydendambaev VD 2014 Content and fatty acid composition of neutral acylglycerols in *Euonymus* fruits. Journal of the American Oil Chemists’ Society 91, 805–814.

[CIT0031] Siloto RM, Truksa M, Brownfield D, Good AG, Weselake RJ 2009 Directed evolution of acyl-CoA:diacylglycerol acyltransferase: development and characterization of *Brassica napus* DGAT1 mutagenized libraries. Plant Physiology and Biochemistry 47, 456–461. 19195902 10.1016/j.plaphy.2008.12.019

[CIT0032] Tjellström H, Strawsine M, Silva J, Cahoon EB, Ohlrogge JB 2013 Disruption of plastid acyl:acyl carrier protein synthetases increases medium chain fatty acid accumulation in seeds of transgenic Arabidopsis. FEBS Letters 587, 936–942. 23454211 10.1016/j.febslet.2013.02.021

[CIT0033] Tran TNT, Shelton J, Brown S, Durrett TP 2017 Membrane topology and identification of key residues of *Ea*DAcT, a plant MBOAT with unusual substrate specificity. The Plant Journal 92, 82–94. 28715115 10.1111/tpj.13636

[CIT0034] Valeri D, Meirelles AJA 1997 Viscosities of fatty acids, triglycerides, and their binary mixtures. Journal of the American Oil Chemists’ Society 74, 1221–1226.

[CIT0035] van Erp H, Bates PD, Burgal J, Shockey J, Browse J 2011 Castor phospholipid:diacylglycerol acyltransferase facilitates efficient metabolism of hydroxy fatty acids in transgenic Arabidopsis. Plant Physiology 155, 683–693. 21173026 10.1104/pp.110.167239 PMC3032459

[CIT0036] Voelker TA, Hayes TR, Cranmer AM, Turner JC, Davies HM 1996 Genetic engineering of a quantitative trait: metabolic and genetic parameters influencing the accumulation of laurate in rapeseed. The Plant Journal 9, 229–241.

[CIT0037] Zhang M, Fan J, Taylor DC, Ohlrogge JB 2009 *DGAT1* and *PDAT1* acyltransferases have overlapping functions in *Arabidopsis* triacylglycerol biosynthesis and are essential for normal pollen and seed development. The Plant Cell 21, 3885–3901. 20040537 10.1105/tpc.109.071795 PMC2814504

