# Supplementary material for: Towards the synthetic design of camelina oil enriched in tailored acetyl-triacylglycerols with medium-chain fatty acids
Source: J Exp Bot. 2018 Jul 6;69(18):4395–402. doi: 10.1093/jxb/ery225 (PMC6093318; doi:10.1093/jxb/ery225)
Supplement: Supplementary figures S1-S2 [file ery225_suppl_supplementary_figures_s1-s2.pdf]

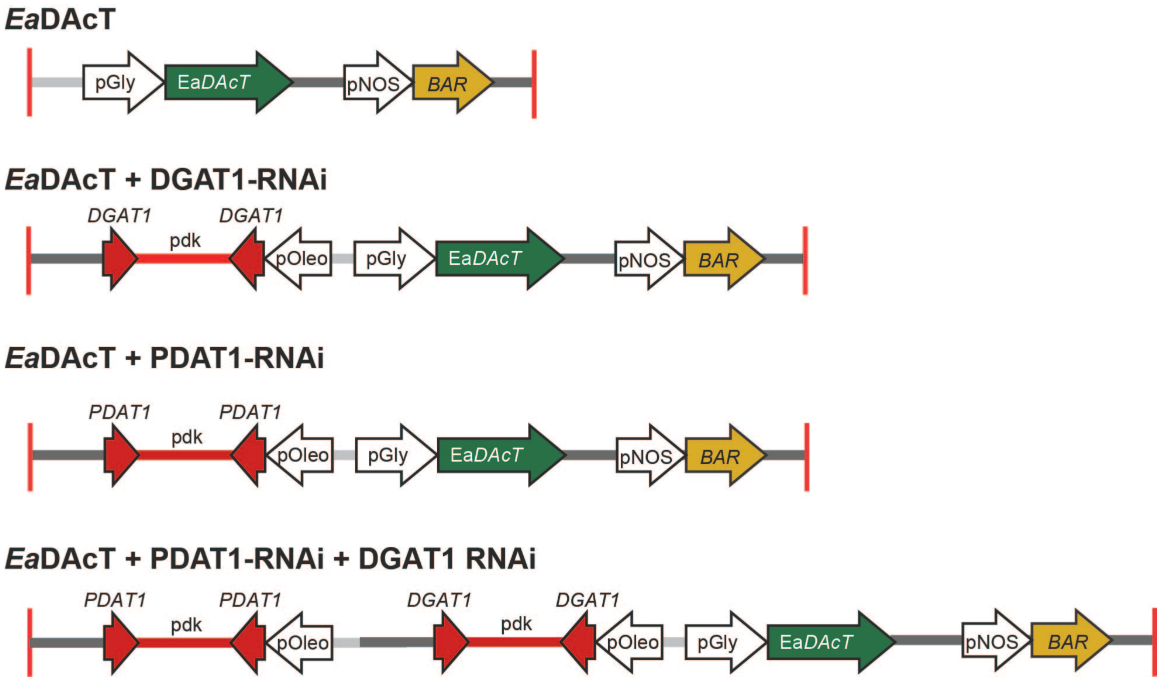


**Figure S1. Constructs used to express *Ea*DAcT and suppress camelina acyltransferases.** Shown are the regions flanked by T-DNA borders (vertical red lines). Promoter regions are indicated by white arrows, terminator sequences are represented by dark gray lines. *DGAT1*, portion of camelina *DGAT1* gene; *BAR*, *Streptomyces hygroscopicus* phosphinothricin acetyltransferase; *Ea*DAcT, native *Ea*DAcT coding sequence; *PDAT1*, portion of camelina *PDAT1* gene; pdk, pdk intron from pHANNIBAL; pGly, soybean glycinin promoter; pNOS, nopaline synthase prometer; pOleo, *Brassica napus* oleosin promoter.

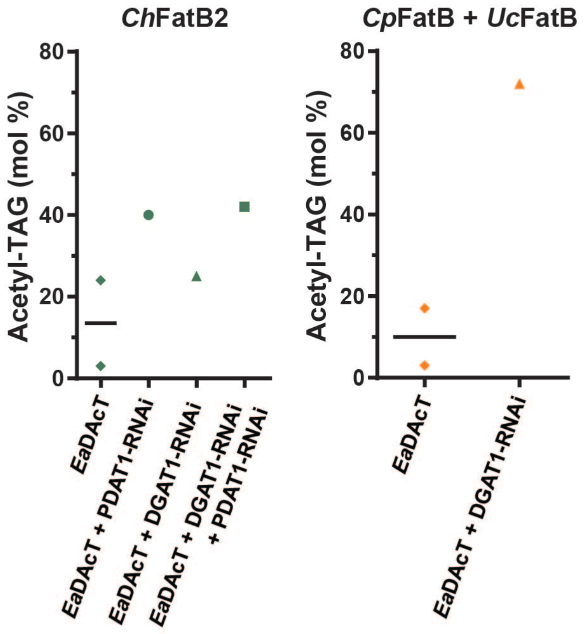


**Figure S2. Expression of *Ea*DAcT combined with down-regulation of DGAT1 enhances acetyl-TAG accumulation.** Scatter plots of the distribution of acetyl-TAG composition of homozygous $T_3$ seeds from independent camelina lines expressing *Ch*FatB2 or *Cp*FatB and *Uc*FatB, and transformed with *Ea*DAcT alone or in combination with RNAi constructs targeting camelina *DGAT1* and *PDAT1* homeologues. Horizontal lines represent the mean values for each sample group.
